# Mechanism of xanthine oxidase in flap ischemia-reperfusion injury and advances in targeted therapy: a mini review

**DOI:** 10.3389/fphys.2025.1705704

**Published:** 2025-11-24

**Authors:** Weidong Jia, Xin Wei, Xu Gong

**Affiliations:** Department of Hand and Foot Surgery, Orthopedics Center, First Hospital of Jilin University, Changchun, China

**Keywords:** ischemia-reperfusion injury, xanthine oxidase, flap, allopurinol, reactive oxygen species

## Abstract

Ischemia-reperfusion injury in flaps refers to a cascade of pathophysiological reactions that aggravate tissue damage or even cause necrosis. During the period of ischemia followed by restored blood reperfusion, a burst of reactive oxygen species is produced. The prevention of flap ischemia-reperfusion injury remains a critical and challenging focus in current research. Xanthine oxidase serves as a major source of reactive oxygen species during ischemia-reperfusion. Allopurinol and febuxostat, xanthine oxidase inhibitor, primarily exerts its protective effects by inhibiting the activity of xanthine oxidase and reducing reactive oxygen species generation, thereby suppressing oxidative stress damage. Additionally, it may improve flap survival through other mechanisms, such as modulating inflammatory responses and suppressing apoptosis. This article systematically reviews the pathological mechanisms and therapeutic advances of skin flap ischemia-reperfusion injury, with a focus on exploring the role of xanthine oxidase inhibitors in flap protection by targeting and regulating oxidative stress pathways, aiming to provide new therapeutic strategies and theoretical basis for clinical prevention and treatment of skin flap ischemia-reperfusion injury.

## 1 Introduction

Flap transplantation is a widely applied surgical technique for repairing skin, subcutaneous soft tissue, or deeper structures such as nerves, tendons, and bones damaged by trauma, tumors, or burns. It plays a critical role in restoring both appearance and function. However, ischemia–reperfusion (I/R) injury is a major contributor to flap failure. In a survey of 1,142 cases, the average survival rate of free flap transplantation exceeded 90%. Nevertheless, 82% of flaps experienced circulatory impairment within the first 24 h, and 9.9% required secondary revascularization. In a large follow-up study of 1,258 free flaps, Chiu et al. reported an 11.9% re-exploration rate, with 58% of cases showing vascular pedicle thrombosis. Of these, only ∼30% were successfully salvaged by thrombectomy and vascular re-anastomosis (Chiu et al., 2017). The lack of timely and effective monitoring and intervention—whether in free or pedicled flaps—remains a major limitation. Postoperative blood supply can be compromised by vasospasm, tissue edema-induced compression, pedicle torsion, or thrombosis. Even when revascularization is achieved, secondary I/R injury often undermines success (Dan Dunn et al., 2015), aggravating tissue damage or causing flap necrosis, with profound effects on prognosis and quality of life. Therefore, preventing and mitigating I/R injury is essential for improving flap survival, functional recovery, and overall patient outcomes.

### 1.1 Pathophysiological mechanisms of flap ischemia-reperfusion injury

Ischemia–reperfusion injury represents a cascade of pathological events involving metabolic dysfunction, cellular damage, and inflammation. During ischemia, oxygen and nutrient deprivation impair mitochondrial oxidative phosphorylation, causing a sharp decline in adenosine triphosphate (ATP) production. Cells switch to anaerobic glycolysis, leading to plenty of lactate accumulation and metabolic acidosis. Concurrent energy depletion disrupts Na^+^/K^+^ and Ca^2+^ pumps, promotes phospholipid degradation, and increases free radical generation. These processes cause cellular swelling, increased membrane permeability, loss of membrane integrity (Mittal et al., 2014), and leakage of intracellular contents (Wu et al., 2018). Additionally, ischemia reduces endothelial nitric oxide (NO) production while increasing vasoconstrictors such as endothelin, thereby inducing vasospasm (Dominguez et al., 2021).

**FIGURE 1:**
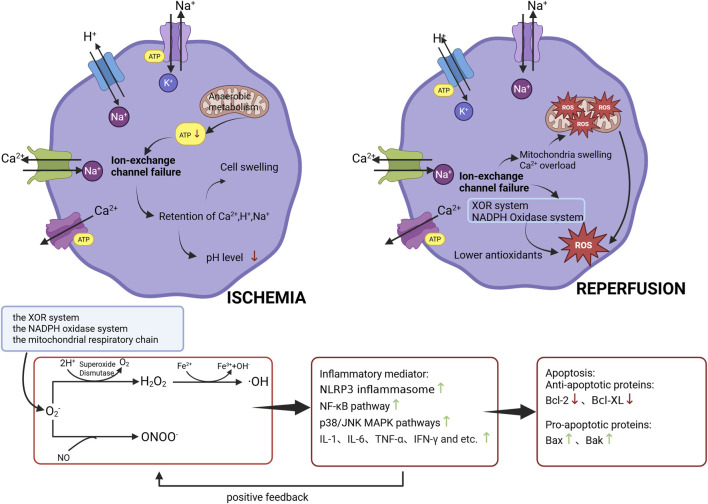
Diagram illustrating cellular changes during ischemia and reperfusion. In ischemia, ion-exchange channel failure, anaerobic metabolism, reduced ATP, cell swelling, and pH drop occur. In reperfusion, calcium overload, mitochondrial swelling, and reactive oxygen species formation from pathways for the XOR system, NADPH oxidase system, and mitochondrial respiratory chain lead to lower antioxidants. The subsequent transformation of superoxide anion. ROS activate inflammation, and apoptosis, with markers expression like NLRP3 inflammasome, NF-κB, p38/JNK MAPK pathways, and proteins such as Bcl-2, Bax, and Bak.

After ischemia, reperfusion further exacerbates injury. Restoration of blood flow, combined with mitochondrial dysfunction and ionic imbalance, activates the xanthine oxidoreductase (XOR) system, producing large amounts of reactive oxygen species (ROS). This oxidative burst triggers mitochondrial injury, calcium overload, leukocyte infiltration, and inflammatory responses (Granger and Kvietys, 2015). ROS—including superoxide anion (O_2_^−^), hydroxyl radical (·OH), hydrogen peroxide (H_2_O_2_), lipid peroxyl radical (LOO·), peroxynitrite (ONOO^−^) and so on—are normally involved in physiological processes such as signal transduction, metabolism, and cell proliferation. However, excessive ROS drive oxidative stress, damaging lipids, proteins, and DNA. ROS also activate inflammatory pathways, promoting leukocyte recruitment and pro-inflammatory cytokine release (Kalogeris et al., 2012). The interplay of oxidative stress and inflammation ultimately results in cell death and tissue injury, contributing to multiple disease processes. Overview of the mechanism of ischemia-reperfusion injury and the subsequent transformation of superoxide anion is illustrated in Figure 1 (Choi et al., 2015).

### 1.2 Pathways of oxygen free radical production and following inflammatory responses and apoptosis and necrosis during intracellular ischemia–reperfusion

Among the major ROS sources described, the xanthine oxidoreductase system, the NADPH oxidase (Nox) family, and the mitochondrial respiratory chain are considered primary mediators of I/R-induced oxidative stress. These pathways represent priority therapeutic targets in organ and tissue I/R injury. While all three enzymatic systems are broadly expressed, the dominant ROS source likely varies among tissues (Wu et al., 2018). Following reperfusion, aforesaid ROS activate multiple inflammatory factors, including the NLRP3 inflammasome, NF-κB, and the p38/JNK MAPK pathways etc. Inflammatory mediators are released in large quantities, amplifying oxygen radical generation and inflammatory responses in a feed-forward cycle of tissue damage. Excessive ROS and inflammation further promote apoptosis and necrosis, exacerbating organ injury and, in severe cases, leading to widespread tissue necrosis (Jurc et al., 2022; Güler et al., 2023).

#### 1.2.1 Xanthine oxidoreductase system

Xanthine oxidoreductase (XOR), a member of the molybdenum–iron–sulfur flavin hydroxylase family, is widely distributed across multiple organs, including the liver, intestine, lung, kidney, heart, brain, and plasma (Wu et al., 2018). XOR exists in two interconvertible forms: xanthine oxidase (XO) and xanthine dehydrogenase (XDH) (Wang et al., 2008). These enzymatic systems transfer electrons from xanthine to oxygen or NAD^+^, respectively, generating O_2_^−^, H_2_O_2_, and NADH (Berry and Hare, 2004). The hypothesis that XO is a major source of ROS in I/R injury was first proposed by Downey JM in 1990 to explain the increased vascular permeability observed after 1 hour of low-flow ischemia followed by reperfusion in the cat small intestine (Granger et al., 1981). XO-derived ROS appear within minutes of reperfusion, whereas neutrophil infiltration (activating the NADPH oxidase system) and mitochondrial dysfunction-mediated ROS generation typically require several hours (Harrison, 2002). Ono T et al. further demonstrated that superoxide generation markedly increased during the early phase of I/R and was significantly attenuated by allopurinol (Ono et al., 2009), confirming XO as the predominant source of ROS at reperfusion onset. This rapid ROS burst directly induces lipid peroxidation and activates neutrophils (via NADPH oxidase) and the complement system, establishing a vicious cycle of “oxidation–inflammation” (Granger, 2025). Accordingly, XOR inhibition disrupts the early ROS surge and prevents downstream tissue injury.

#### 1.2.2 NADPH oxidase system

The Nox/Duox family of NADPH oxidases represents another important source of ROS in I/R (Lassègue and Griendling, 2010). These enzymes are widely expressed across cell types, including vascular cells, with significant mRNA and protein expression documented in multiple tissues. Nox/Duox enzymes are established contributors to ROS production under diverse pathological conditions. Their role in reperfusion injury is supported by two key observations (Chiu et al., 2017): upregulated expression and activity of Nox in ischemic tissues, and (Dan Dunn et al., 2015) attenuation of ROS generation and I/R-induced injury following pharmacological inhibition or genetic suppression of Nox expression (Yokota et al., 2011).

#### 1.2.3 Mitochondrial oxidative respiratory chain

Mitochondria constitute a third major ROS source during I/R. Mitochondrial respiratory chain–derived ROS were first reported in 1966 (Jensen, 1966). Chance et al. subsequently demonstrated that isolated mitochondria could generate H_2_O_2_ (Chance et al., 1979), later shown to arise from superoxide dismutation within mitochondria (Forman and Kennedy, 1974). ROS production primarily occurs at Complex I (NADH dehydrogenase) and Complex III (ubiquinol–cytochrome c reductase) of the respiratory chain.

### 1.3 The influnce of endogenous antioxidant defense systems during ischemia–reperfusion injury

The body maintains a sophisticated antioxidant defence system that relies on endogenous enzymatic and nonenzymatic antioxidants. The role of the main defense antioxidants which basically include superoxide dismutase (SOD), catalase (CAT) and glutathione peroxidase (GPX) is important and indispensable to resist damaging effects from relevant reactive oxidative substance in the entire defense strategy of antioxidants, especially in reference to super oxide anion radical (O_2_^−^) which is continuously produced during normal metabolic processes (Ighodaro and Akinloye, 2018; Jomova et al., 2024).

## 2 Mechanism and therapeutic advances of xanthine oxidase inhibitors in flap ischemia-reperfusion injury

### 2.1 Pharmacological effects of xanthine oxidase inhibitors

Currently, the clinically available XO inhibitors include allopurinol and febuxostat.

Allopurinol, metabolized *in vivo* to oxypurinol, competitively inhibits XO, blocking the conversion of hypoxanthine and xanthine to uric acid. Febuxostat, in contrast, binds directly to the molybdenum pterin center at the XO active site, suppressing enzymatic activity and simultaneously reducing both uric acid and ROS production. Both agents are approved for treating acute and chronic hyperuricemia and related disorders (Nishino and Okamoto, 2015). Beyond urate lowering, they exert protective effects in various models of I/R injury (Ullah et al., 2020; Sun Guifang, 2019; Yajuan, 2019; Zhang et al., 2021; Shin et al., 2015; Wang et al., 2015; Tsuda et al., 2012). Allopurinol not only suppresses ROS production and dampens inflammation but also facilitates ATP regeneration through the purine salvage pathway by sparing hypoxanthine, thereby ameliorating energy metabolism disturbances (Frenguelli, 2019). Febuxostat, approved in 2009, has shown promising protective efficacy in animal I/R models. However, large-scale clinical evidence—particularly regarding long-term, multi-organ outcomes—remains limited. Moreover, concerns regarding elevated cardiovascular risk necessitate cautious use, especially in elderly patients and those with multiple comorbidities (Bruce, 2006). Notably, XO inhibitors act primarily at the extracellular surface of vascular endothelial cells, where XO is concentrated, making them more accessible via the bloodstream.

### 2.2 Therapeutic applications and advances of xanthine oxidase inhibitors in flap ischemia-reperfusion injury

#### 2.2.1 Reduction of ROS generation and attenuation of oxidative stress

**FIGURE 2:**
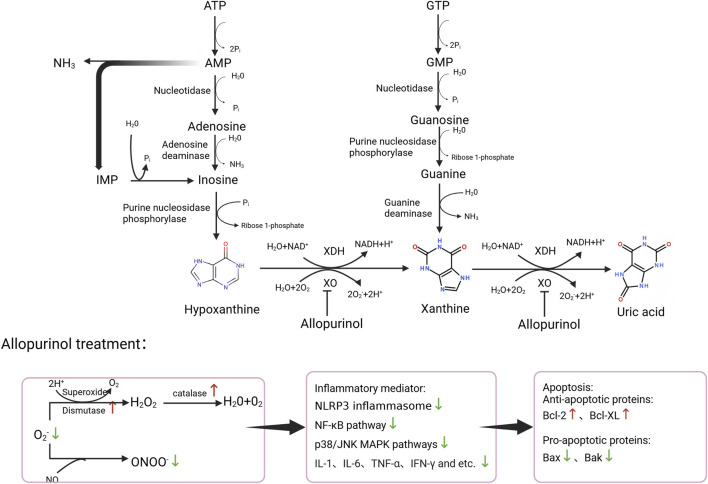
Metabolic pathway diagram showing the conversion of ATP and GTP with xanthine dehydrogenase (XDH) or xanthine oxidase (XO) into uric acid via intermediates like hypoxanthine and xanthine and mechanisms of action of XO inhibitors.

The functional dynamics of the xanthine oxidoreductase (XOR) system are illustrated in Figure 2. Under physiological conditions, hypoxanthine is oxidized by xanthine dehydrogenase (XDH) in the presence of H_2_O and NAD^+^, producing xanthine, NADH, and H^+^. XDH further catalyzes the conversion of xanthine to uric acid, again generating NADH and H^+^. This pathway does not directly generate reactive oxygen species (ROS), as electrons are efficiently transferred to NAD^+^. Reactive oxygen species (ROS) are catalyzed by superoxide dismutase (SOD) to form hydrogen peroxide, which is subsequently decomposed into non-toxic water and oxygen by catalase (Ighodaro and Akinloye, 2018). During ischemia–reperfusion (I/R), however, ATP degradation in the ischemic phase leads to marked accumulation of hypoxanthine. Concurrently, intracellular Ca^2+^ overload activates Ca^2+^-dependent proteases that convert XDH to xanthine oxidase (XO) through oxidation of critical cysteine residues and/or limited proteolysis. Upon reperfusion, when oxygen supply is restored, XO utilizes molecular oxygen as the terminal electron acceptor to catalyze hypoxanthine and xanthine oxidation into uric acid. Moreover, suppression or impairment of the antioxidant system, along with excessive generation of ROS, results in substantial depletion of antioxidant enzymes such as SOD, CAT, and GPX. Consequently, released large amounts of O_2_^−^ and H_2_O_2_ further gives rise to more toxic reactive species, including ·OH, LOO· and ONOO^−^ (Ighodaro and Akinloye, 2018). The resulting ROS directly oxidize amino acid residues, induce conformational alterations, and promote protein cross-linking, thereby impairing enzymatic function. They can also damage nucleic acids by oxidizing bases, breaking the deoxyribose backbone, or disrupting base-pairing, which interferes with replication and transcription. Furthermore, ROS accelerate telomere attrition and drive cellular senescence (Cecarini et al., 2007).

Cytokines including interleukin-1 (IL-1), interferon-γ (IFN-γ), interleukin-6 (IL-6), and tumor necrosis factor-α (TNF-α) (Dopp et al., 2011; Schwartz et al., 1995) exacerbate this process by upregulating XDH/XO mRNA expression, enhancing XOR activity (Mittal et al., 2014; Dopp et al., 2011; Schwartz et al., 1995; Falciani et al., 1992; Poss et al., 1996; Pfeffer et al., 1994), and promoting the XDH-to-XO shift (Falciani et al., 1992; Vorbach et al., 2003). The activated XOR system, in turn, drives massive ROS release and further cytokine production (Berry and Hare, 2004; Xiaotao et al., 2024; Yapca et al., 2013).

Experimental studies support the protective role of XO inhibition in flap survival. Prada FS et al. reported that allopurinol increased the survival area of rat inferior epigastric artery perforator flaps (Prada et al., 2002), while Mehdi Rasti Ardakani et al. demonstrated reduced necrosis in abdominal flaps in dogs following allopurinol treatment (Ardakani, 2017). Collectively, these findings suggest a beneficial effect of allopurinol in improving flap viability.

Mechanistically, Guansong Wang et al. showed that hypoxia activates XDH/XO via the IL-6–JAK/STAT pathway in pulmonary vascular endothelial cells (Wang et al., 2008). Allopurinol, as an XO inhibitor, suppresses IL-6 expression (Prieto-Moure et al., 2017; Gitaswari et al., 2024), thereby dampening IL-6–JAK/STAT–mediated XO activation, reducing ROS production (Wang et al., 2008), and attenuating inflammatory signaling.

In addition, Zhang Y.S. et al. identified the XO–VPO1 axis as a novel oxidative stress pathway involving XO and vascular peroxidase 1 (VPO1), a heme-containing peroxidase. Activation of this pathway drives excessive ROS production, including hypochlorous acid (HOCl), H_2_O_2_, and O_2_^−^, thereby exacerbating cellular damage. Allopurinol inhibits this axis by reducing XO activity, suppressing XO-derived H_2_O_2_ and uric acid, and downregulating VPO1 expression at both the mRNA and protein levels (Zhang et al., 2021). Moreover, allopurinol upregulates endogenous antioxidant defenses, such as glutathione and catalase (Sagor et al., 2015; Milcheski et al., 2013). Superoxide (O_2_^−^) generated during I/R also reacts rapidly with nitric oxide (NO), a critical vasoprotective molecule that maintains vasodilation, suppresses smooth muscle proliferation, limits platelet aggregation, and prevents leukocyte adhesion. This reaction yields peroxynitrite (ONOO^−^), depleting NO bioavailability, impairing vasodilation, and inducing endothelial dysfunction. Oxypurinol, the active metabolite of allopurinol, has been shown to counteract this effect (Cardillo et al., 1997).

Further evidence from Gitaswari I’s group indicates that during flap I/R, allopurinol reduces ROS by inhibiting XO, suppresses NF-κB activation, downregulates pro-inflammatory cytokines (IL-6, TNF-α), and upregulates vascular endothelial growth factor (VEGF) (Gitaswari et al., 2024). Increased VEGF expression enhances NO and endothelin release (Yi et al., 2020), thereby promoting angiogenesis, vasodilation, and microcirculatory recovery. Collectively, these effects significantly improve flap survival (Gitaswari et al., 2024).

Kang HB et al. reported that oxypurinol, the active metabolite of allopurinol, induces the expression of heme oxygenase-1 (HO-1) (Kang et al., 2023). HO-1 catalyzes the degradation of cytotoxic free heme in the presence of O_2_ and NADPH oxidase, producing biliverdin, Fe^2+^, and carbon monoxide (CO). Biliverdin is subsequently converted to bilirubin, which exerts potent antioxidant effects by neutralizing reactive oxygen species (ROS) (Huang et al., 2022; Chen et al., 2020). Simultaneously, reduced NADPH oxidase activity limits O_2_^−^ production, promotes Fe^2+^ sequestration by ferritin, and suppresses the Fenton reaction (Fujii et al., 2010; Oh et al., 2013). CO mediates additional protective effects: it inhibits NF-κB nuclear translocation, thereby reducing pro-inflammatory cytokines (TNF-α, IL-6) and adhesion molecules (ICAM-1, VCAM-1), which collectively limit leukocyte infiltration (Araujo et al., 2012). Moreover, CO activates the soluble guanylate cyclase–cGMP pathway, enhancing vasodilation and improving tissue perfusion (Chen et al., 2015; Vera et al., 2005). Bilirubin also inhibits activation of the NLRP3 inflammasome, further curbing inflammation (Zhang et al., 2025; Li et al., 2022; Vítek, 2020).

Soliman E et al. demonstrated that allopurinol mitigates ischemia–reperfusion (I/R) injury by modulating the xanthine oxidase (XO)–peroxisome proliferator-activated receptor γ (PPAR-γ) signaling pathway (Soliman et al., 2023). PPAR-γ regulates oxidative stress by suppressing pro-oxidant genes such as NADPH oxidase (Soliman et al., 2023) and preserving mitochondrial structure and function (Yeligar et al., 2018), thereby reducing ROS generation. This lowers NO oxidation into peroxynitrite (ONOO^−^) and concurrently enhances antioxidant enzyme expression, including superoxide dismutase and glutathione peroxidase (De Nuccio et al., 2020; Yongyue and Yingjing, 2020). PPAR-γ activation also inhibits NF-κB signaling and downregulates transcription of pro-inflammatory cytokines (TNF-α, IL-6, and IL-1β) (Bernardo and Minghetti, 2006). In addition, it promotes cell survival by upregulating anti-apoptotic proteins (Bcl-2) and suppressing pro-apoptotic proteins (Bax) (Wu et al., 2021; Wang et al., 2023). Mechanistically, allopurinol inhibits XO, reduces ROS production, and upregulates PPAR-γ, creating a feedback loop that attenuates oxidative stress and inflammation, ultimately protecting tissues and organs.

#### 2.2.2 Inhibition of inflammatory response

During I/R, ROS activate multiple inflammatory cascades, including the NLRP3 inflammasome, NF-κB, and the p38/JNK MAPK pathways. Terada et al. observed that neutrophils exacerbate endothelial injury through the release of reactive oxygen metabolites (O_2_^−^, H_2_O_2_, HOCl), whereas allopurinol suppresses neutrophil adhesion to endothelial cells (Granger and Kvietys, 2015; Ichikawa et al., 1997). Beyond direct ROS inhibition, allopurinol reduces XO-dependent signaling, thereby indirectly blocking NF-κB activation (Guzik et al., 2025). This significantly downregulates transcription of IL-6, IL-1β, and TNF-α (Choi et al., 2015; Shin et al., 2015; Gitaswari et al., 2024; Shenkar and Abraham, 1997; Bahriz et al., 2025). Excessive activation of the IL-6–JAK/STAT3 axis further promotes apoptosis and endothelial barrier dysfunction; allopurinol alleviates this by lowering IL-6 expression and STAT3 phosphorylation (Prieto-Moure et al., 2017). Likewise, I/R-induced activation of the JNK/p38 MAPK pathway, which drives inflammatory mediator release and stress responses, is effectively suppressed by allopurinol (Shin et al., 2015; Kang et al., 2023; Kuanlu and Yong, 2010).

Zhou J. Qiao et al. showed that ROS accumulation during I/R induces cellular damage and promotes the release of high-mobility group box 1 (HMGB1). HMGB1 activates the TLR4/NF-κB pathway, driving TNF-α and IL-6 release, upregulating pro-apoptotic proteins (Bax, Caspase-3), and downregulating the anti-apoptotic protein Bcl-2. Allopurinol reduces ROS generation, thereby suppressing HMGB1 activity, alleviating inflammation and apoptosis, and ultimately mitigating organ injury (Zhou et al., 2016). Similarly, Ives A. et al. demonstrated that XO promotes mitochondrial ROS production via the PI3K–AKT–mTOR pathway, which activates the NLRP3 inflammasome. Allopurinol, as an XO inhibitor, markedly suppresses IL-1β secretion, thereby limiting inflammasome assembly and dampening the inflammatory response (Ives et al., 2015).

#### 2.2.3 Anti-apoptotic effects

Apoptosis induced by oxidative stress occurs through multiple mechanisms, including mitochondrial dysfunction, activation of intracellular signaling pathways, and DNA damage. During reperfusion, the sudden influx of oxygen causes excessive production of reactive oxygen species (ROS) and intracellular Ca^2+^ overload, leading to the opening of the mitochondrial permeability transition pore (mPTP). This event results in mitochondrial membrane depolarization, matrix swelling, and outer membrane rupture, allowing pro-apoptotic factors such as cytochrome c to escape into the cytoplasm, thereby activating downstream apoptotic cascades. Membrane depolarization also contributes to ATP depletion, further promoting cell death (Choi et al., 2015; Pizzino et al., 2017; Wang et al., 2011). Recent studies confirm that ROS can induce mPTP opening during reperfusion, precipitating apoptosis (Lee and Lee, 2006). Allopurinol mitigates this process by reducing ROS generation, indirectly stabilizing the mitochondrial membrane, preserving ATP synthesis, and decreasing the release of pro-apoptotic factors through the mPTP, thus suppressing downstream apoptotic signaling (Choi et al., 2015; Brandão et al., 2018).

Among the major signaling pathways activated by oxidative stress, the p53 pathway plays a central role by upregulating pro-apoptotic proteins such as Bax and downregulating anti-apoptotic proteins such as Bcl-2 (Shin et al., 2015). Wang S. et al. demonstrated that febuxostat enhances the expression of anti-apoptotic proteins Bcl-2 and Bcl-XL, reduces pro-apoptotic proteins Bax and Bak, and increases the Bcl-2/Bax ratio (Wang et al., 2015). Other pathways implicated in oxidative stress-induced apoptosis include JNK and ERK. Ju-Hyun Shin et al. reported that during ischemia–reperfusion (I/R), pro-apoptotic signals such as JNK and p38 are upregulated and phosphorylated, whereas survival signals such as ERK are suppressed. Allopurinol reversed this pattern by markedly inhibiting JNK and p38 activation, attenuating the JNK-mediated suppression of Bcl-2, and increasing both the expression and phosphorylation of Bcl-2. In addition, allopurinol reduced Bax expression, thereby restoring the Bcl-2/Bax balance (Shin et al., 2015).

Oxidative stress also induces DNA damage, activating DNA damage response pathways and triggering apoptosis (Schumacker, 2006; Lin and Beal, 2006). ROS-mediated DNA damage promotes the formation of necrotic bodies via poly (ADP-ribose) polymerase (PARP) activation. Excessive PARP-1 activity depletes ATP stores and drives necrotic cell death (Ryu et al., 2010).

## 3 Discussion

Current evidence indicates that the enzymatic reactions underlying ischemia–reperfusion injury mediated by the xanthine oxidoreductase (XOR) system involve only xanthine oxidase (XO) and xanthine dehydrogenase. In the renal ischemia/reperfusion (I/R) model, both the XOR inhibitor (Soliman et al., 2023) and the knockout of the XOR gene in mice (Haga et al., 2017) significantly attenuate oxidative stress and inflammation, thereby improving renal function. In the cardiac I/R model, XO inhibitors suppress ROS production, preserve catalase activity, and mitigate myocardial injury (Brown et al., 1988). In the skeletal muscle I/R model, inhibition of the XOR system effectively alleviates oxidative stress in skeletal muscle (Kuroda et al., 2020; Paradis et al., 2016). Similarly, in the hepatic I/R model, XOR inhibitors reduce ROS generation by preventing xanthine accumulation during ischemia, eliminating free radicals. (Tang et al., 2022; Cannistrà et al., 2016; Singh et al., 2023). Collectively, evidence about organ I/R models highlight the pivotal role of the XO system in the burst of oxidative stress and comprehensively demonstrate the therapeutic potential of XO inhibitors, such as allopurinol, in preclinical I/R injury studies. In contemporary clinical research, XO inhibitors have shown promising efficacy in attenuating oxidative stress during I/R across multiple organs, including the heart, kidneys, and brain. Clinical trials in improving thrombolysis in myocardial infarction flow grades among patients with acute ST-segment elevation myocardial infarction following percutaneous coronary intervention (KermaniAlghoraishi et al., 2023), assessing long-term neurodevelopmental outcomes of neonates with hypoxic-ischemic encephalopathy (Maiwald et al., 2019) and in preventing contrast-induced nephropathy among patients undergoing percutaneous coronary intervention (Mansoor et al., 2021; Sarhan et al., 2023), substantial evidence indicates that xanthine oxidase inhibitors effectively have a positive impact. However, clinical evidence in flap I/R models remains lacking. Furthermore, investigating combination strategies that pair XO inhibition with other therapeutic modalities may offer synergistic protection for tissues and organs. Potential synergistic strategies include physical interventions (e.g., shockwave therapy, transcutaneous electrical stimulation) and pharmacological approaches, such as antioxidants (Chaves et al., 2020) (e.g., N-acetylcysteine, vitamin C),NADPH oxidase inhibitors (e.g., GKT137831) and mitochondria-protective agents (Ross and Benjamin, 2025) (e.g., SS-31/elamipretide). Such combination therapies may simultaneously suppress ROS generation at its source through XO inhibition while attenuating secondary mitochondrial ROS amplification. The NADPH oxidase system and the mitochondrial respiratory chain participate in I/R-related redox reactions through more complex mechanisms, with limited effective drug options and unresolved safety concerns. Additionally, experimental data suggest that Nox4 deficiency may aggravate oxidative stress injury (Schröder et al., 2012; Nlandu-Khodo et al., 2016), whereas allopurinol does not interfere with the protective Nox4–Nrf2 signaling axis. Moreover, integrating XO inhibitors with immuno-inflammatory-targeted therapies (e.g., NLRP3 or IL-1 inhibitors) may provide more comprehensive protection by suppressing both oxidative stress and downstream inflammatory cascades, thereby reducing reperfusion injury and delayed inflammatory necrosis more effectively (Zhang et al., 2024).

Taken together, XOR represents the earliest and most accessible source of ROS in I/R, offering a precise, clinically feasible, and safe therapeutic target. Inhibiting XOR disrupts the initial oxidative burst, reduces the release of inflammatory mediators while modulating autophagy and apoptosis and interrupts the subsequent “oxidation–inflammation–apoptosis” cascade. As the only antioxidant target consistently shown to be effective across multiple organ systems, XOR inhibition holds considerable promise for clinical translation. This review highlights the therapeutic relevance of XOR and its inhibitors in flap ischemia–reperfusion injury.

## References

[B1] Araujo J. A. Zhang M. Yin F. (2012). Heme oxygenase-1, oxidation, inflammation, and atherosclerosis. Front. Pharmacol. 3, 119. 10.3389/fphar.2012.00119 22833723 PMC3400084

[B2] Ardakani M. R. (2017). Effect of systemic antioxidant allopurinol therapy on skin flap survival. Adv. Plast. Reconstr. Surg. 1. 10.31700/2572-6684.000110 PMC5339610 28289614

[B3] Bahriz H. A. Abdelaziz R. R. El-Kashef D. H. (2025). Allopurinol abates hepatocellular carcinoma in rats via modulation of NLRP3 inflammasome and NF-κB pathway. Schmiedeb. Arch. Pharmacol. 398, 6043–6058. 10.1007/s00210-024-03666-8 39636403

[B4] Bernardo A. Minghetti L. (2006). PPAR-γ agonists as regulators of microglial activation and brain inflammation. Curr. Pharm. Des. 12, 93–109. 10.2174/138161206780574579 16454728

[B5] Berry C. E. Hare J. M. (2004). Xanthine oxidoreductase and cardiovascular disease: molecular mechanisms and pathophysiological implications. J. Physiol. 555, 589–606. 10.1113/jphysiol.2003.055913 14694147 PMC1664875

[B6] Brandão R. I. Gomes R. Z. Lopes L. Linhares F. S. Vellosa J. C. R. Paludo K. S. (2018). Remote post-conditioning and allopurinol reduce ischemia-reperfusion injury in an infra-renal ischemia model. Ther. Adv. Cardiovasc Dis. 12, 341–349. 10.1177/1753944718803309 30295166 PMC6266249

[B7] Brown J. M. Terada L. S. Grosso M. A. Whitmann G. J. Velasco S. E. Patt A. (1988). Xanthine oxidase produces hydrogen peroxide which contributes to reperfusion injury of ischemic, isolated, perfused rat hearts. J. Clin. Invest 81, 1297–1301. 10.1172/JCI113448 3127425 PMC329662

[B8] Bruce S. P. (2006). Febuxostat: a selective xanthine oxidase inhibitor for the treatment of hyperuricemia and gout. Ann. Pharmacother. 40, 2187–2194. 10.1345/aph.1H121 17132810

[B9] Cannistrà M. Ruggiero M. Zullo A. Gallelli G. Serafini S. Maria M. (2016). Hepatic ischemia reperfusion injury: a systematic review of literature and the role of current drugs and biomarkers. Int. J. Surg. 33, S57–S70. 10.1016/j.ijsu.2016.05.050 27255130

[B10] Cardillo C. Kilcoyne C. M. Cannon R. O. Quyyumi A. A. Panza J. A. (1997). Xanthine oxidase inhibition with oxypurinol improves endothelial vasodilator function in hypercholesterolemic but not in hypertensive patients. Hypertension 30, 57–63. 10.1161/01.HYP.30.1.57 9231821

[B11] Cecarini V. Gee J. Fioretti E. Amici M. Angeletti M. Eleuteri A. M. (2007). Protein oxidation and cellular homeostasis: emphasis on metabolism. Biochim. Biophys. Acta (BBA) - Mol. Cell Res. 1773, 93–104. 10.1016/j.bbamcr.2006.08.039 17023064

[B12] Chance B. Sies H. Boveris A. (1979). Hydroperoxide metabolism in mammalian organs. Physiol. Rev. 59, 527–605. 10.1152/physrev.1979.59.3.527 37532

[B13] Chaves C. N. Kiyomi Koike M. Jacysyn J. Rasslan R. Azevedo Cerqueira A. Pereira C. S. (2020). N-acetylcysteine reduced ischemia and reperfusion damage associated with steatohepatitis in mice. Int. J. Mol. Sci. 21, 4106. 10.3390/ijms21114106 32526845 PMC7313069

[B14] Chen H.-H. Lu P.-J. Chen B.-R. Hsiao M. Ho W.-Y. Tseng C.-J. (2015). Heme oxygenase-1 ameliorates kidney ischemia-reperfusion injury in mice through extracellular signal-regulated kinase 1/2-enhanced tubular epithelium proliferation. Biochim. Biophys. Acta (BBA) - Mol. Basis Dis. 1852, 2195–2201. 10.1016/j.bbadis.2015.07.018 26232688

[B15] Chen Z. Vong C. T. Gao C. Chen S. Wu X. Wang S. (2020). Bilirubin nanomedicines for the treatment of reactive oxygen species (ROS)-mediated diseases. Mol. Pharm. 17, 2260–2274. 10.1021/acs.molpharmaceut.0c00337 32433886

[B16] Chiu Y.-H. Chang D.-H. Perng C.-K. (2017). Vascular complications and free flap salvage in head and neck reconstructive surgery: analysis of 150 cases of reexploration. Ann. Plast. Surg. 78, S83–S88. 10.1097/SAP.0000000000001011 28166137

[B17] Choi E. K. Jung H. Kwak K. H. Yeo J. Yi S. J. Park C. Y. (2015). Effects of allopurinol and apocynin on renal ischemia-reperfusion injury in rats. Transpl. Proc. 47, 1633–1638. 10.1016/j.transproceed.2015.06.007 26293026

[B18] Dan Dunn J. Alvarez L. A. Zhang X. Soldati T. (2015). Reactive oxygen species and mitochondria: a nexus of cellular homeostasis. Redox Biol. 6, 472–485. 10.1016/j.redox.2015.09.005 26432659 PMC4596921

[B19] De Nuccio C. Bernardo A. Troiano C. Brignone M. S. Falchi M. Greco A. (2020). NRF2 and PPAR-γ pathways in oligodendrocyte progenitors: focus on ROS protection, mitochondrial biogenesis and promotion of cell differentiation. Int. J. Mol. Sci. 21, 7216. 10.3390/ijms21197216 33003644 PMC7583077

[B20] Dominguez S. Varfolomeev E. Brendza R. Stark K. Tea J. Imperio J. (2021). Genetic inactivation of RIP1 kinase does not ameliorate disease in a mouse model of ALS. Cell Death Differ. 28, 915–931. 10.1038/s41418-020-00625-7 32994544 PMC7937687

[B21] Dopp J. M. Philippi N. R. Marcus N. J. Olson E. B. Bird C. E. Moran J. J. M. (2011). Xanthine oxidase inhibition attenuates endothelial dysfunction caused by chronic intermittent hypoxia in rats. Respiration 82, 458–467. 10.1159/000329341 21846958 PMC3214835

[B36] Falciani F. Ghezzi P. Terao M. Cazzaniga G. Garattini E. (1992). Interferons induce xanthine dehydrogenase gene expression in L929 cells. Biochem. J. 285, 1001–1008. 10.1042/bj2851001 1379796 PMC1132894

[B22] Forman H. J. Kennedy J. A. (1974). Role of superoxide radical in mitochondrial dehydrogenase reactions. Biochem. Biophys. Res. Commun. 60, 1044–1050. 10.1016/0006-291X(74)90418-5 4372996

[B23] Frenguelli B. G. (2019). The purine salvage pathway and the restoration of cerebral ATP: implications for brain slice physiology and brain injury. Neurochem. Res. 44, 661–675. 10.1007/s11064-017-2386-6 28836168 PMC6420432

[B24] Fujii M. Inoguchi T. Sasaki S. Maeda Y. Zheng J. Kobayashi K. (2010). Bilirubin and biliverdin protect rodents against diabetic nephropathy by downregulating NAD(P)H oxidase. Kidney Int. 78, 905–919. 10.1038/ki.2010.265 20686447

[B25] Gitaswari I. Hamid A. R. R. H. Sanjaya I. G. P. H. Samsarga G. W. Lestari A. A. W. Niryana I. W. (2024). Systemic allopurinol administration reduces malondialdehyde, interleukin 6, tumor necrosis factor α, and increases vascular endothelial growth factor in random flap wistar rats exposed to nicotine. Acta Chir. Plast. 66, 60–66. 10.48095/ccachp202460 39174340

[B26] Granger D. N. (2025). Role of xanthine oxidase and granulocytes in ischemia-reperfusion injury. 10.1152/ajpheart.1988.255.6.H1269 3059826

[B27] Granger D. N. Kvietys P. R. (2015). Reperfusion injury and reactive oxygen species: the evolution of a concept. Redox Biol. 6, 524–551. 10.1016/j.redox.2015.08.020 26484802 PMC4625011

[B28] Granger D. N. Rutili G. McCord J. M. (1981). Superoxide radicals in feline intestinal ischemia. Gastroenterology 81, 22–29. 10.1016/0016-5085(81)90648-X 6263743

[B29] Güler M. C. Tanyeli A. Ekinci Akdemir F. N. Eraslan E. Özbek Şebin S. Güzel Erdoğan D. (2023). An overview of ischemia–reperfusion injury: review on oxidative stress and inflammatory response. Eurasian J. Med. 54, S62–S65. 10.5152/eurasianjmed.2022.22293 36655447 PMC11163358

[B30] Guzik T. J. Olszanecki R. Sadowski J. Kapelak B. Rudzi P. Jopek A. (2025). Superoxide dismutase activity and expression in human venous and arterial bypass graft vessels. 15985711

[B31] Haga Y. Ohtsubo T. Murakami N. Noguchi H. Kansui Y. Goto K. (2017). Disruption of xanthine oxidoreductase gene attenuates renal ischemia reperfusion injury in mice. Life Sci. 182, 73–79. 10.1016/j.lfs.2017.06.011 28625358

[B32] Harrison R. (2002). Structure and function of xanthine oxidoreductase: where are we now? Free Radic. Biol. Med. 33, 774–797. 10.1016/s0891-5849(02)00956-5 12208366

[B33] Huang Y. Liu Y. Yu S. Li W. Li J. Zhao B. (2022). Biliverdin reductase a protects lens epithelial cells against oxidative damage and cellular senescence in age‐related cataract. Oxid. Med. Cell Longev. 2022, 5628946. 10.1155/2022/5628946 35910837 PMC9325611

[B34] Ichikawa H. Flores S. Kvietys P. R. Wolf R. E. Yoshikawa T. Granger D. N. (1997). Molecular mechanisms of anoxia/reoxygenation-induced neutrophil adherence to cultured endothelial cells. Circ. Res. 81, 922–931. 10.1161/01.RES.81.6.922 9400372

[B35] Ighodaro O. M. Akinloye O. A. (2018). First line defence antioxidants-superoxide dismutase (SOD), catalase (CAT) and glutathione peroxidase (GPX): their fundamental role in the entire antioxidant defence grid. Alex. J. Med. 54, 287–293. 10.1016/j.ajme.2017.09.001

[B37] Ives A. Nomura J. Martinon F. Roger T. LeRoy D. Miner J. N. (2015). Xanthine oxidoreductase regulates macrophage IL1β secretion upon NLRP3 inflammasome activation. Nat. Commun. 6, 6555. 10.1038/ncomms7555 25800347 PMC4382995

[B38] Jensen P. K. (1966). Antimycin-insensitive oxidation of succinate and reduced nicotinamide-adenine dinucleotide in electron-transport particles I. pH dependency and hydrogen peroxide formation. Biochim. Biophys. Acta (BBA) 122, 157–166. 10.1016/0926-6593(66)90057-9 4291041

[B39] Jomova K. Alomar S. Y. Alwasel S. H. Nepovimova E. Kuca K. Valko M. (2024). Several lines of antioxidant defense against oxidative stress: antioxidant enzymes, nanomaterials with multiple enzyme-mimicking activities, and low-molecular-weight antioxidants. Arch. Toxicol. 98, 1323–1367. 10.1007/s00204-024-03696-4 38483584 PMC11303474

[B40] Jurcau A. Ardelean A. I. (2022). Oxidative stress in ischemia/reperfusion injuries following acute ischemic stroke. Biomedicines 10, 574. 10.3390/biomedicines10030574 35327376 PMC8945353

[B41] Kalogeris T. Baines C. P. Krenz M. Korthuis R. J. (2012). “Cell biology of ischemia/reperfusion injury,” in International review of cell and molecular biology. Elsevier, 229–317. 10.1016/B978-0-12-394309-5.00006-7 PMC3904795 22878108

[B42] Kang H. B. Lim C. K. Kim J. Han S. J. (2023). Oxypurinol protects renal ischemia/reperfusion injury via heme oxygenase-1 induction. Front. Med. 10, 1030577. 10.3389/fmed.2023.1030577 36968831 PMC10033620

[B43] KermaniAlghoraishi M. D. M. Sanei H. HeshmatGhahdarijani K. Ghahramani R. Honarvar M. Sadeghi M. (2023). Impact of allopurinol pretreatment on coronary blood flow and revascularization outcomes after percutaneous coronary intervention in acute STEMI patients: a randomized double blind clinical trial. ARYA Atheroscler. J. 19, 1–9. 10.48305/arya.2023.11577.2121 38883850 PMC11178989

[B44] Kuanlu F. Yong X. (2010). Effects of allopurinol on p38MAPK activation and apoptosis in ischemic-reperfused myocardium. Jilin Med. J. 31, 5699–5701. Available online at: https://kns.cnki.net/kcms2/article/abstract?v=IKKGlZ0AkeX0v26ITbLvo-wbDHqLp_6e12dPxz9bV1J94tGI16gc7F0NsvRITTbgDzE9CJQVNwpTEo-E75Gv2h6I_11wq5OKlJcN3seJWqx3jnJB66oifympwEvz1wjQcQAQHX0TkaKuUrS5qksALxbLkx-eQC1NU11ysj-6D-_Df_ NpPEHtA==&uniplatform=NZKPT&language=CHS.

[B45] Kuroda Y. Togashi H. Uchida T. Haga K. Yamashita A. Sadahiro M. (2020). Oxidative stress evaluation of skeletal muscle in ischemia–reperfusion injury using enhanced magnetic resonance imaging. Sci. Rep. 10, 10863. 10.1038/s41598-020-67336-4 32616815 PMC7331576

[B46] Lassègue B. Griendling K. K. (2010). NADPH oxidases: functions and pathologies in the vasculature. Thromb. Vasc. Biol. 30, 653–661. 10.1161/atvbaha.108.181610 19910640 PMC2841695

[B47] Lee W.-Y. Lee S.-M. (2006). Synergistic protective effect of ischemic preconditioning and allopurinol on ischemia/reperfusion injury in rat liver. Biochem. Biophys. Res. Commun. 349, 1087–1093. 10.1016/j.bbrc.2006.08.140 16959212

[B48] Li Y. Sheng H. Yan Z. Guan B. Qiang S. Qian J. (2022). Bilirubin stabilizes the mitochondrial membranes during NLRP3 inflammasome activation. Biochem. Pharmacol. 203, 115204. 10.1016/j.bcp.2022.115204 35944727

[B49] Lin M. T. Beal M. F. (2006). Mitochondrial dysfunction and oxidative stress in neurodegenerative diseases. Nature 443, 787–795. 10.1038/nature05292 17051205

[B50] Maiwald C. A. Annink K. V. Rüdiger M. Benders MJNL Van Bel F. Allegaert K. (2019). Effect of allopurinol in addition to hypothermia treatment in neonates for hypoxic-ischemic brain injury on neurocognitive outcome (ALBINO): study protocol of a blinded randomized placebo-controlled parallel group multicenter trial for superiority (phase III). BMC Pediatr. 19, 210. 10.1186/s12887-019-1566-8 31248390 PMC6595623

[B51] Mansoor K. Suliman M. Amro M. Malik S. Amro A. Curtis Z. (2021). Protective effect of allopurinol in preventing contrast-induced nephropathy among patients undergoing percutaneous coronary intervention: a systematic review and meta-analysis. Arch. Med. Sci. – Atheroscler. Dis. 6, 196–202. 10.5114/amsad.2021.112226 36161220 PMC9487829

[B53] Milcheski D. A. Nakamoto H. A. Tuma P. Nóbrega L. Ferreira M. C. (2013). Experimental model of degloving injury in rats: effect of allopurinol and pentoxifylline in improving viability of avulsed flaps. Ann. Plast. Surg. 70, 366–369. 10.1097/SAP.0b013e318230601a 21921788

[B54] Mittal M. Siddiqui M. R. Tran K. Reddy S. P. Malik A. B. (2014). Reactive oxygen species in inflammation and tissue injury. Antioxid. Redox Signal. 20, 1126–1167. 10.1089/ars.2012.5149 23991888 PMC3929010

[B55] Nishino T. Okamoto K. (2015). Mechanistic insights into xanthine oxidoreductase from development studies of candidate drugs to treat hyperuricemia and gout. JBIC, J. Biol. Inorg. Chem. 20, 195–207. 10.1007/s00775-014-1210-x 25501928 PMC4334109

[B56] Nlandu-Khodo S. Dissard R. Hasler U. Schäfer M. Pircher H. Jansen-Durr P. (2016). NADPH oxidase 4 deficiency increases tubular cell death during acute ischemic reperfusion injury. Sci. Rep. 6, 38598. 10.1038/srep38598 27924932 PMC5141508

[B57] Oh S. W. Lee E. S. Kim S. Na K. Y. Chae D. W. Kim S. (2013). Bilirubin attenuates the renal tubular injury by inhibition of oxidative stress and apoptosis. BMC Nephrol. 14, 105. 10.1186/1471-2369-14-105 23683031 PMC3681641

[B58] Ono T. Tsuruta R. Fujita M. Aki H. S. Kutsuna S. Kawamura Y. (2009). Xanthine oxidase is one of the major sources of superoxide anion radicals in blood after reperfusion in rats with forebrain ischemia/reperfusion. Brain Res. 1305, 158–167. 10.1016/j.brainres.2009.09.061 19781528

[B59] Paradis S. Charles A.-L. Meyer A. Lejay A. Scholey J. W. Chakfé N. (2016). Chronology of mitochondrial and cellular events during skeletal muscle ischemia-reperfusion. Am. J. Physiol. Cell Physiol. 310, C968–C982. 10.1152/ajpcell.00356.2015 27076618 PMC4935201

[B60] Pfeffer K. D. Huecksteadt T. P. Hoidal J. R. (1994). Xanthine dehydrogenase and xanthine oxidase activity and gene expression in renal epithelial cells. Cytokine and steroid regulation. J. Immunol. 153, 1789–1797. 10.4049/jimmunol.153.4.1789 8046245

[B61] Pizzino G. Irrera N. Cucinotta M. Pallio G. Mannino F. Arcoraci V. (2017). Oxidative stress: harms and benefits for human health. Oxid. Med. Cell Longev. 2017, 8416763. 10.1155/2017/8416763 28819546 PMC5551541

[B62] Poss W. B. Huecksteadt T. P. Panus P. C. Freeman B. A. Hoidal J. R. (1996). Regulation of xanthine dehydrogenase and xanthine oxidase activity by hypoxia. Am. J. Physiol. 270, L941–L946. 10.1152/ajplung.1996.270.6.L941 8764218

[B63] Prada F. S. Arrunategui G. Alves M. C. Ferreira M. C. Zumiotti A. V. (2002). Effect of allopurinol, superoxide‐dismutase, and hyperbaric oxygen on flap survival. Microsurgery 22, 352–360. 10.1002/micr.10073 12497572

[B64] Prieto-Moure B. Lloris-Carsí J. M. Belda-Antolí M. Toledo-Pereyra L. H. Cejalvo-Lapeña D. (2017). Allopurinol protective effect of renal ischemia by downregulating TNF-α, IL-1β, and IL-6 response. J. Invest Surg. 30, 143–151. 10.1080/08941939.2016.1230658 27690698

[B65] Ross G. R. Benjamin I. J. (2025). Antioxidants in cardiovascular health: implications for disease modeling using cardiac organoids. Antioxidants 14, 1202. 10.3390/antiox14101202 41154511 PMC12561675

[B66] Ryu H.-C. Kim C. Kim J.-Y. Chung J.-H. Kim J.-H. (2010). UVB radiation induces apoptosis in keratinocytes by activating a pathway linked to “BLT2-Reactive Oxygen Species.”. J. Invest Dermatol 130, 1095–1106. 10.1038/jid.2009.436 20090768

[B52] Sagor M.A.T. Tabassum N. Potol M. A. Alam M. A. (2015). Xanthine oxidase inhibitor, allopurinol, prevented oxidative stress, fibrosis, and myocardial damage in isoproterenol induced aged rats. Oxid. Med. Cell Longev. 2015, 1–9. 10.1155/2015/478039 26137187 PMC4475550

[B67] Sarhan I. I. Abdellatif Y. A. Saad R. E. Teama N. M. (2023). Renoprotective effect of febuxostat on contrast-induced acute kidney injury in chronic kidney disease patients stage 3: randomized controlled trial. BMC Nephrol. 24, 65. 10.1186/s12882-023-03114-4 36949408 PMC10035112

[B68] Schröder K. Zhang M. Benkhoff S. Mieth A. Pliquett R. Kosowski J. (2012). Nox4 is a protective reactive oxygen species generating vascular NADPH oxidase. Circ. Res. 110, 1217–1225. 10.1161/CIRCRESAHA.112.267054 22456182

[B69] Schumacker P. T. (2006). Reactive oxygen species in cancer cells: live by the sword, die by the sword. Cancer Cell 10, 175–176. 10.1016/j.ccr.2006.08.015 16959608

[B70] Schwartz M. D. Repine J. E. Abraham E. (1995). Xanthine oxidase-derived oxygen radicals increase lung cytokine expression in mice subjected to hemorrhagic shock. Am. J. Respir. Cell Mol. Biol. 12, 434–440. 10.1165/ajrcmb.12.4.7695923 7695923

[B71] Shenkar R. Abraham E. (1997). Hemorrhage induces rapid *in vivo* activation of CREB and NF-kappaB in murine intraparenchymal lung mononuclear cells. Am. J. Respir. Cell Mol. Biol. 16, 145–152. 10.1165/ajrcmb.16.2.9032121 9032121

[B72] Shin J.-H. Chun K. S. Na Y.-G. Song K.-H. Kim S. I. Lim J.-S. (2015). Allopurinol protects against ischemia/reperfusion-induced injury in rat urinary bladders. Oxid. Med. Cell Longev. 2015, 906787–906788. 10.1155/2015/906787 26491537 PMC4600567

[B73] Singh K. Gupta J. K. Kumar S. Anurag M. S. Patel A. (2023). Hepatic ischemia-reperfusion injury: protective approaches and treatment. Curr. Mol. Pharmacol. 17, e030823219400. 10.2174/1874467217666230803114856 37534481

[B74] Soliman E. Elshazly S. M. Shewaikh S. M. El-shaarawy F. (2023). Reno- and hepato-protective effect of allopurinol after renal ischemia/reperfusion injury: crosstalk between xanthine oxidase and peroxisome proliferator-activated receptor gamma signaling. Food Chem. Toxicol. 178, 113868. 10.1016/j.fct.2023.113868 37269893

[B75] Sun Guifang W. X. (2019). Therapeutic effects and mechanisms of allopurinol in rats with chronic heart failure. J. Med. Res. 48, 73–76. Available online at: https://kns.cnki.net/kcms2/article/abstract?v=IKKGlZ0AkeVMFWl8ghJhzmZfgyQ4GFA0ZauR_AYCYOjgt1nHVU29HOzPT3DI_S5zaHfPxixusqHI7mzK8p6wdMC-2PfWRClsqI7Ta4HV_AJhCTwn1vcqh8Ll7Luv68KjGctuywWIl-ZNy2PFZKJkXOCQq0gkvz39WuyR14eLEWTpT18paYkyrudHkJIMjNCB&uniplatform=NZKPT&language=CHS.

[B76] Tang S. Mao X. Chen Y. Yan L. Ye L. Li S. (2022). Reactive oxygen species induce fatty liver and ischemia-reperfusion injury by promoting inflammation and cell death. Front. Immunol. 13, 870239. 10.3389/fimmu.2022.870239 35572532 PMC9098816

[B77] Tsuda H. Kawada N. Kaimori J. Kitamura H. Moriyama T. Rakugi H. (2012). Febuxostat suppressed renal ischemia–reperfusion injury via reduced oxidative stress. Biochem. Biophys. Res. Commun. 427, 266–272. 10.1016/j.bbrc.2012.09.032 22995295

[B78] Ullah W. Khanal S. Khan R. Basyal B. Munir S. Minalyan A. (2020). Efficacy of allopurinol in cardiovascular diseases: a systematic review and meta-analysis. Cardiol. Res. 11, 226–232. 10.14740/cr1066 32595807 PMC7295562

[B79] Vera T. Henegar J. R. Drummond H. A. Rimoldi J. M. Stec D. E. (2005). Protective effect of carbon monoxide–releasing compounds in ischemia-induced acute renal failure. J. Am. Soc. Nephrol. 16, 950–958. 10.1681/ASN.2004090736 15728782

[B80] Vítek L. (2020). Bilirubin as a signaling molecule. Med. Res. Rev. 40, 1335–1351. 10.1002/med.21660 32017160

[B81] Vorbach C. Harrison R. Capecchi M. R. (2003). Xanthine oxidoreductase is central to the evolution and function of the innate immune system. Trends Immunol. 24, 512–517. 10.1016/S1471-4906(03)00237-0 12967676

[B82] Wang G. Qian P. Jackson F. R. Qian G. Wu G. (2008). Sequential activation of JAKs, STATs and xanthine dehydrogenase/oxidase by hypoxia in lung microvascular endothelial cells. Int. J. Biochem. and Cell Biol. 40, 461–470. 10.1016/j.biocel.2007.08.008 17920330 PMC2276459

[B83] Wang W. Z. Baynosa R. C. Zamboni W. A. (2011). Update on ischemia-reperfusion injury for the plastic surgeon: 2011. Plast. Reconstr. Surg. 128, 685e–692e. 10.1097/PRS.0b013e318230c57b 22094770

[B84] Wang S. Li Y. Song X. Wang X. Zhao C. Chen A. (2015). Febuxostat pretreatment attenuates myocardial ischemia/reperfusion injury via mitochondrial apoptosis. J. Transl. Med. 13, 209. 10.1186/s12967-015-0578-x 26136232 PMC4489215

[B85] Wang S. Cai Y. Bu R. Wang Y. Lin Q. Chen Y. (2023). PPARγ regulates macrophage polarization by inhibiting the JAK/STAT pathway and attenuates myocardial ischemia/reperfusion injury *in vivo* . Cell Biochem. Biophys. 81, 349–358. 10.1007/s12013-023-01137-0 37129843

[B86] Wu M.-Y. Yiang G.-T. Liao W.-T. Tsai A. P.-Y. Cheng Y.-L. Cheng P.-W. (2018). Current mechanistic concepts in ischemia and reperfusion injury. Cell Physiol. Biochem. 46, 1650–1667. 10.1159/000489241 29694958

[B87] Wu L. Yu Q. Cheng P. Guo C. (2021). PPARγ plays an important role in acute hepatic ischemia-reperfusion injury via AMPK/mTOR pathway. PPAR Res. 2021, 6626295–15. 10.1155/2021/6626295 34285690 PMC8275421

[B88] Xiaotao W. Bijli K. M. Kleinhenz J. M. (2024). Research progress on the effects of reactive oxygen species on ischemia-reperfusion injury flaps and the intervention of traditional Chinese medicine. J. Math. Med. 37, 287–292. Available online at: https://kns.cnki.net/kcms2/article/abstract?v=TajfHsrud9-y4XKQMIyJ70XapPbHHueqz4EVoOF558VwI6wkxKB-KTLAtdAfTuxRKyfICcQIxV8pzankQDyh4ZWzGA3MsBJ2fOiJfgxV9lj7OyAhbwUqpPx5oL1nW_Z7SdncnoMAt1JLB42hFmnyai79lRo1umnmz3uEcI6KIu-VfH7cVvqCy5eONiKPgXrv&uniplatform=NZKPT&language=CHS.

[B89] Yajuan Y. (2019). “Allopurinol, a xanthine oxidase inhibitor, improves oxidative stress-mediated atrial remodeling in diabetic animals,”. Tianjin: Tianjin Medical University. 10.27366/d.cnki.gtyku.2019.000208

[B90] Yapca O. E. Borekci B. Suleyman H. (2013). Ischemia-reperfusion damage. Eurasian J. Med. 45, 126–127. 10.5152/eajm.2013.24 25610264 PMC4261480

[B91] Yeligar S. M. Kang B.-Y. Bijli K. M. Kleinhenz J. M. Murphy T. C. Torres G. (2018). PPARγ regulates mitochondrial structure and function and human pulmonary artery smooth muscle cell proliferation. Am. J. Respir. Cell Mol. Biol. 58, 648–657. 10.1165/rcmb.2016-0293OC 29182484 PMC5946324

[B92] Yi D. Borekci B. Suleyman H. (2020). Protective effect of allopurinol against renal injury in rats with chronic intermittent hypoxia. Chin. Contemp. Med. 27, 18–22. Available online at: https://kns.cnki.net/kcms2/article/abstract?v=IKKGlZ0AkeVaIR3G3uD5dcjNbsE4_ZVOhtTWR7dWGOXTDogdhUocndHGJxkDoOoEUjjJ7FL7xOokfRUz8lHTW=zgkicwveIadH3L2l9GJjqsy5_KH0jMSXnMlXFmaKMuJGg5MgfgxW45khraUJHrUijEo-F2hYTwZCPeyTJt95fuohyqT-C6lw59z6rUywUUf&uniplatform=NZKPT&language=CHS.

[B93] Yokota H. Narayanan S. P. Zhang W. Liu H. Rojas M. Xu Z. (2011). Neuroprotection from retinal ischemia/reperfusion injury by NOX2 NADPH oxidase deletion. Investig. Opthalmology Vis. Sci. 52, 8123–8131. 10.1167/iovs.11-8318 21917939 PMC3208002

[B94] Yongyue H. Yingjing S. (2020). Effects of allopurinol's inhibition of xanthine oxidase on heart rate variability in hypoxic and hyperoxic rats. Guangdong Med. J. 41, 14–18. 10.13820/j.cnki.gdyx.20191344

[B95] Zhang Y.-S. Lu L.-Q. Jiang Y.-Q. Li N.-S. Luo X.-J. Peng J.-W. (2021). Allopurinol attenuates oxidative injury in rat hearts suffered ischemia/reperfusion via suppressing the xanthine oxidase/vascular peroxidase 1 pathway. Eur. J. Pharmacol. 908, 174368. 10.1016/j.ejphar.2021.174368 34302816

[B96] Zhang M. Liu Q. Meng H. Duan H. Liu X. Wu J. (2024). Ischemia-reperfusion injury: molecular mechanisms and therapeutic targets. Signal Transduct. Target. Ther. 9, 12. 10.1038/s41392-023-01688-x 38185705 PMC10772178

[B97] Zhang Y. Luan H. Song P. (2025). Bilirubin metabolism and its application in disease prevention: mechanisms and research advances. Inflamm. Res. 74, 81. 10.1007/s00011-025-02049-w 40413269

[B98] Zhou J. Qiu T. Zhang L. Chen Z. Wang Z. Ma X. (2016). Allopurinol preconditioning attenuates renal ischemia/reperfusion injury by inhibiting HMGB1 expression in a rat model. Acta Cir. Bras. 31, 176–182. 10.1590/S0102-865020160030000005 27050788

